# Understanding the Gap Between Nursing Workforce in the United States and Population Needs—A Policy Brief

**DOI:** 10.1055/s-0043-1775724

**Published:** 2023-09-27

**Authors:** Mayar Al Mohajer

**Affiliations:** 1 Department of Medicine, Baylor College of Medicine, Houston, Texas, United States

**Keywords:** nursing, policy, shortage

## Abstract

**Purpose** This report is intended to analyze the root causes for the current gap between the nursing workforce and population needs in the United States. It aims to consolidate what is known about these contributing reasons and provide evidence-based recommendations for action.

**Methods** The report utilized the Sample, Phenomenon of Interest, Design, Evaluation, Research type framework to develop the research question and the 5 Whys methodology for the root cause analysis.

**Results** This report highlighted six major causative problems, including workforce market mismatch, poor financing design, inadequate governance, flawed technologies, insufficient research, and suboptimal service delivery. A detailed evaluation of root causes with supported evidence is presented.

**Conclusion** The report provided seven actionable recommendations based on the analysis: (1) strengthening the nursing role in advancing equity, (2) investing in nursing well-being, (3) changing policies and payment structure, (4) including nursing in technology design, (5) strengthening nursing education, (6) developing a robust public health emergencies preparedness plan, and (7) investing in relevant research.

## Introduction

**Fig. 1:**
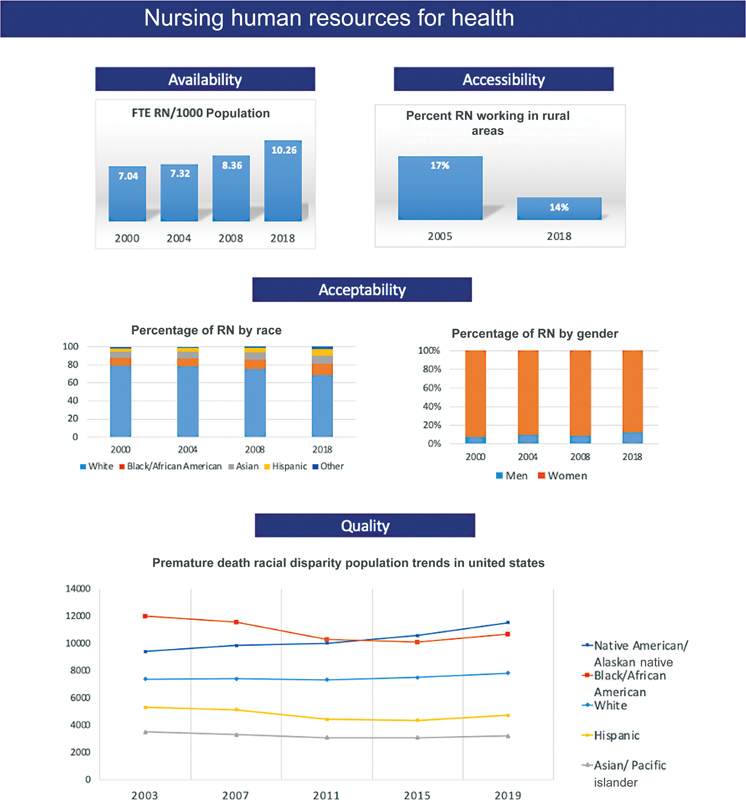
This figure shows different aspects of nursing human resources for health including availability, accessibility, acceptability, and quality. Data adapted from Wakefield et al and America's Health Rankings. 6 7 FTE: full-time equivalent; RN: registered nurse.

Nursing is a crucial part of the U.S. health workforce, and without it, achieving the health system outcomes, such as accessibility, quality, and efficiency, 1 would not be possible. This was echoed in the World Health Organization (WHO) report titled: “A Universal Truth: No Health Without A Workforce” 2 . Although the current staffing of 102.6 FTE/10,000 population exceeds the WHO target benchmark of 59.4 (2), there exists a considerable and worsening shortage of over 1 million nurses 3 due to an unexpected shift of market forces from equilibrium. 4 In addition to nursing availability, other domains of the workforce, including accessibility, acceptability, and quality 5 6 7 are affected ( Fig. 1 ).

**Fig. 2:**
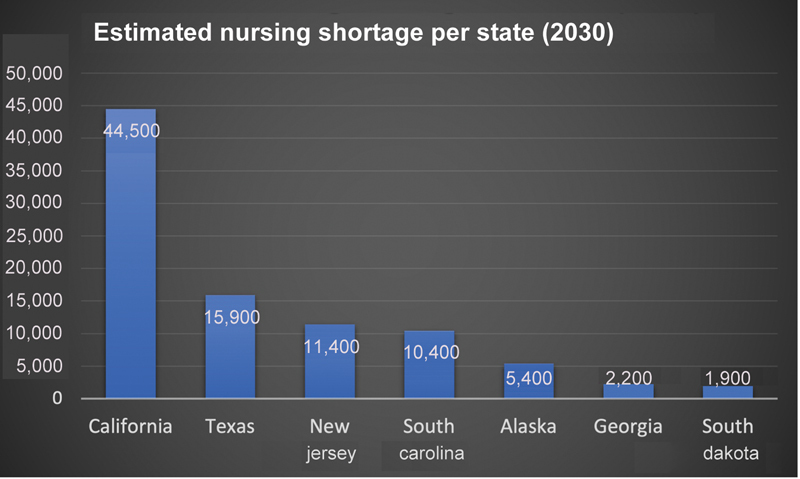
This figure shows the states with highest estimated nursing shortage. Data adapted from USAHS. 9

There are several contextual factors 8 that can explain the gap between the nursing workforce in the U.S. and population needs. These include structural factors (aging population, geography 9 [ Fig. 2 ], social determinants of health (SDOH), health inequity, and nursing student loans), situational (coronavirus disease 2019 [COVID-19] and opioid crisis) and cultural factors (traditional view of nursing as a women's job), and international factors (global nursing shortage). 10

This report outlines a root cause analysis (RCA) to analyze the causes of the aforementioned gap. Then, it will present policy recommendations based on the result of this RCA.

## Methodology

The 5 Whys methodology 11 was selected for the RCA as it provided an extensive analysis of factors associated with the nursing gap. The Sample, Phenomenon of Interest, Design, Evaluation, Research type format ( Table 1 ) was utilized for evidence synthesis, 12 leading to the following research question: “Using the Five Whys framework (D) of qualitative research (R), what were the root causes (E) of the inability of nursing workforce (PI) to meet population need in the United States (S)?”.

**Table 1:** SPIDER (Sample, Phenomenon of Interest, Design, Evaluation, Research type) format for evidence synthesis 12

Component	Description
Sample (S)	Population in the United States
Phenomenon of interest (PI)	Nursing unable to meet population demand
Design (D)	5 Whys
Evaluation (E)	Problem and root causes
Research type (R)	Qualitative

Note: Data adapted from 12

Note: SPIDER format for evidence synthesis.

A literature review of peer-reviewed studies and gray literature was carried out. Three databases were included Medline, EMBASE, and Web of Science, in addition to gray literature sources encompassing governmental and nongovernmental organization reports.

A keyword strategy was applied, which comprised the following: (“nurs*” OR “healthcare*” OR “hospital?” OR “workforce”) AND (“shortage?” OR “suppl*” OR “demand*”).

Inclusion criteria comprised articles that addressed nursing shortages in the United States and explored their causes. Full-text sources published in English between 2010 and 2021 were assessed, including observational and experimental studies, policy briefs, and commentaries. Studies were excluded if they were abstract, if addressed nursing shortages outside the United States, or if focused on other health care workers.

The selected articles were extracted using a standard form detailing the study design, location, and findings. The author (M.A.) assessed the studies for inclusion. Quality assessment was not performed. The WHO building blocks 13 framework was used for grouping causative factors and evidence synthesis.

## Results

Based on the literature review, 3 4 6 14 15 16 17 18 19 20 21 22 23 24 25 26 a total of 5,043 studies were identified. After excluding duplicates and studies that do not address the nursing shortages in the United States, a total of 245 studies were included. We grouped the causes of the gap between nursing and population needs into the following categories ( Table 2 ).

**Table 2:** Root cause analysis (5 WHYs) for the inability of nursing workforce to meet population needs across the United States

1st Why (causative problem)	2nd Why	3rd Why	4th Why	5th Why (root causes)
Workforce market shift (mismatch between demand and supply)	Increased demand for nursing	Growing population needs nationwide	Increased medical comorbidities	Aging population, obesity, racial inequality, geographic trends, poverty
			Increased mental and behavioral illnesses	substance use, gun violence, anxiety, depression
			Inadequate access to PCP	Low uptake of Medicaid expansion, insurance not mandatory, out-of-pocket fees, NPs scope restriction
			High maternal mortality	Racial inequities, low numbers of hospital providing maternity care
			Worsening PCP shortages	Decreasing hours, retirements, increasing demand, NP scope restriction
		Clinical specialty needs	Geriatric nursing shortage	Geriatric physician shortage (due to low salaries)
			ICU nursing shortage	COVID-19, aging population, increased medical comorbidities, and ECMO
			Psychiatric nursing shortage	Increased in mental health (COVID-19, anxiety, depression, and suicide attempts)
			Dialysis nursing shortage	Aging population, increased hypertension
			Anesthesia nursing shortage	Aging population, increased comorbidities
		Uneven distribution of patient needs	Higher need in rural areas	Lack of transportation, education, and poverty
				Larger physician shortage in rural areas
			Insurance status	No universal coverage, out-of-pocket payments
	Reduced supply of nurses	Increased retirement rates	Large proportion of nurses are Baby Boomers	Shifting U.S. demographics
		Early retirement or changing jobs	Long-term impact of COVID-19	Staff burnout, lack of safety culture, reduced PPE supply
				Shifting roles and responsibilities
				Providing end-of-life care
			Impact of human-caused disasters	Insufficient pandemic preparedness knowledge or skills
				No decision-making authority
				Lack of trust with nursing and healthcare administration
				Scarce resources and staffing shortages
				Feeling unsafe due to gun violence, active shooters in hospitals and terrorism
			Cultural and political factors	Social unrest due to political climate
				Systemic racism
				Underrepresentation and low diversity (gender, race, and ethnicity)
		Insufficient number of graduating nurses	Low recruitment and admissions to nursing schools	Increased tuition and diminished financial support
				Absence of short pathways for graduation
				Absence of distance learning opportunities
				Poverty
			Low retention rates in nursing schools Insufficient nursing training slots	lack of social, emotional, academic, and economic support
				Lack of transparency (passing rate)
				Shortage in nursing faculty (low salaries, lack of diversity, and burnout)
			Inability to adapt to changes in demand (e.g. COVID-19)	Cost of training
				Absence of distance learning
				Inflexible curricula
Poor financing design	Fee-for-service system not advancing equity	Nurses not incentivized for cognitive activities and/or coordination	No CPT codes exist for these services	Technology not incorporated
				Financing systems heavily rely on fee-for-service
		APRN are not credited for diagnoses and treatment	Advanced practitioners can only bill under “incident-to” billing	Medicare reimburses supervising providers rather than actual providers
		Most schools do not have full time nurses	Limitation on billing ability of school nurses	Varying state, school, and local policies
				Complexity and scarcity of funding sources and not taking advantage of currently available ones
		Nursing shortage in public health	Inability to hire vacant nursing positions, offer job security or promotion opportunities	Cut in federal funding by 10% (2010–2019)
				Reduction in local and state funding
Inadequate leadership/ governance response	Government policies not advancing equity	Shortage of staff providing delivery	CNM do not have autonomy for providing birth	State law restriction on providing selected services
		Reduced production capacities of RNs and NPs	State requirement for physician oversight (24 states)	Physicians and public concerns about nursing and NPs abilities to diagnose patients and prescribe medications
		Shortage of mental health providers in rural areas	APRN not empowered to provide treatment for substance use	Federal and state laws prohibiting nonphysicians from prescribing treatment (e.g., buprenorphine)
		Nurses not participating in telehealth	Few opportunities exist to provide telehealth	Unclear if waivers to provide telehealth during the COVID-19 pandemic will continue to exist
	Education system not advancing equity	Insufficient integration of SDOH in nursing education	No expanded opportunities to build competencies	SDOH not prioritized by nursing schools
	Public health policies insufficient for emergency preparedness	Lack of robust national, state and local action plans to address nursing workforce response to disasters	ACA focused on medical and healthcare readiness but ignored nursing preparedness	Nursing preparedness was not a key policy priority
				Lack of metrics to measure facilities preparedness
			Local, state and national policies did not delineate how to equip nurses with skills needed	Nursing education was not prioritized
Flawed medical products/ technologies	Challenges with adaption to new technologies	Stress from charting and reviewing EHR	Significant time spent with EHR	Nursing not included in EHR selection and implementation
				Suboptimal nursing training on EHR and investment in user interface
		Alarm fatigue and missed alarms	Excessive electronic alarms	Nursing not included in alert design
				Poorly designed health informatic policies
		Errors in medication dispensation	Soundalike medications and insufficient time	Lack of safety culture
				Insufficient technology training programs for nursing
	New technologies not person-centered	Population health innovative projects not integrated with nurse practice	Nurses with technology expertise are not included in project design	Nurses are not part of priority setting
Insufficient information and research	Research gaps in the field of nursing	Uncoordinated and fragmented efforts without evidence-based recommendations	Inadequate funding of nursing research	Lack of alignment between funders' priorities and needed research infrastructure
Not optimized service delivery	Reduced health quality	Inability to deliver person-centered care	No emphasis on codesigning interventions and services with the population	Nurses are often not included in developing and optimizing services
		Lack of care coordination	Health system parts are not incentivized to collaborate	Financial healthcare payments are often not bundled
		Ineffective and unsafe nursing care	Limited nursing education and time	Absence of safety culture
			Gaps to achieve cultural humility	Structural racism
				Cultural competence not incorporated in nursing schools
		Nursing care not comprehensive	Several services not fully covered (private nursing, some nursing home and home care)	Limited resources and deficient federal and state policies
	Reduced health equities and accessibility	Nurses not empowered to address SDOH	Nurses not provided with sufficient skills, training, and education	Lack of resources and prioritization for SDOH
		COVID-19 related inequities	Limited ability of nurses to aid in linking health to social and economic needs	State restrictions for nursing scope
				Insufficient nursing time, knowledge and skills

Abbreviations: ACA, Affordable Care Act; APRN, advanced practice registered nurse; CNM, certified nurse midwife, COVID-19, coronavirus disease 2019; CPT, current procedure terminology; ECMO, extracorporeal membrane oxygenation; EHR, electronic health record; ICU, intensive care unit; NP, nurse practitioner; PCP, primary care physicians; PPE, personal protective equipment; SDOH, social determinants of health.

Source: Data adapted from 3 4 6 14 15 16 17 18 19 20 21 22 23 24 25 26

**Fig. 3:**
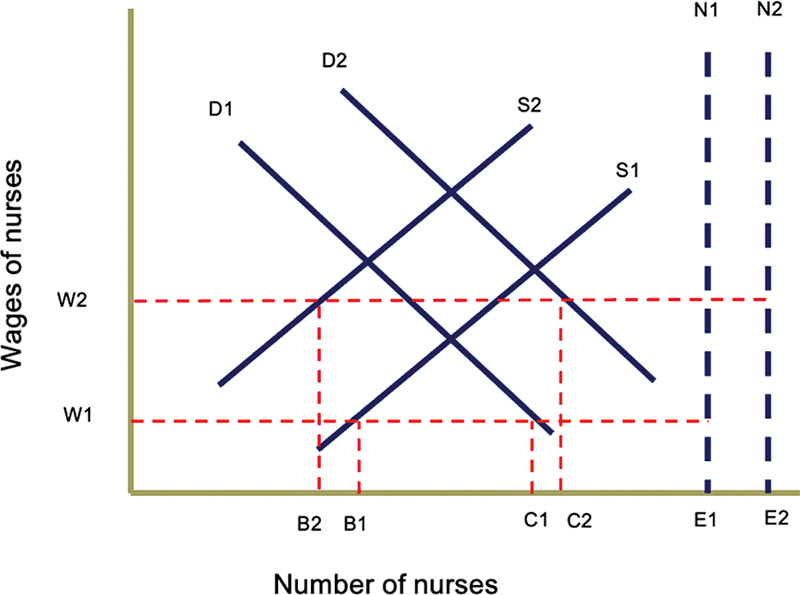
This diagram shows the labor market for nurses before the coronavirus 2019 (COVID-19) pandemic given the parameters (demand D1, supply S1, need N1, and wage W1) leading to a shortage of C1 to B1 to meet the prepandemic demand and E1 to B1 to meet the prepandemic need. During the COVID-19 pandemic, the demand increased to D2, the supply decreased to S2, the need increased to N2, and the wage to W2, leading to a larger shortage of C2 to B2 to the meet the pandemic demand and E2 to B2 to meet the pandemic need. Data adapted from Scheffler et al. 26

1. Workforce market mismatch: The two major causes are increased demand and reduced supply. 26 The high demand stems from structural factors such as growing population needs (aging, substance use, and inadequate access) and situational factors (ICU shortage during the COVID-19 pandemic). The reduced supply resulted from gender and racial underrepresentation (cultural), low rates of graduating nurses, and high retirement rates (structural), which was augmented by the COVID-19 pandemic (situational) due to staff burnout. 17 19 Fig. 3 shows labor market forces before and after the pandemic and how COVID-19 worsened the existing nursing shortage.
2. Poor financing design: The current finance system limits nurses' involvement in patient care by not crediting them for work coordinating services, diagnosis, or management. In addition, cuts in federal funding augmented the public health nursing shortage. 6
3. Inadequate leadership/governance: Existing policies do not support building nursing skills to advance equity, providing telehealth, treating substance abuse, delivering babies, or preparing for pandemics, and they restrict nurses' ability to diagnose and manage patients. 3 18
4. Flawed medical products/technologies: There are several reasons why technology contributed to the nursing gap. Nurses were not included in the design of many projects. This poor design resulted in them spending significant time with electronic health records rather than clinical duties. This was further compounded by the fact that they deal with excessive alarms leading to burnout and stress. In addition, a lack of safety culture and training led to increased medication errors. 22
5. Insufficient information and research: Inadequate funding of nursing research due to lack of prioritizations from funders has led to fragmented and uncoordinated care, which lacked the focus on evidence-based medicine. 23
6. Not optimized service delivery: Service delivery has suffered from reduced quality, equities, and accessibilities. This is caused by not including nurses in service delivery design, not prioritizing cultural competencies in nursing schools, structural racism, absence of safety culture, financial payment models, limited resources, and state restrictions on nursing scope. 24-25

## Discussion

In order to overcome the nursing gap, the following policy recommendations for health system reform were developed ( Table 3 ) based on the RCA above, the Future of Nursing 2020-2030 report 6 and other gray literature publications. 20 27 28

**Table 3:** Tasks required for policy recommendations

Recommendations	Specific tasks	Data collection	Indicators	Goals
Short term (by 2024)				
Investing in health and well-being of nurses	Nursing programs should incorporate materials on nursing well-being in their curricula	Online surveys of nursing programs	Proportion of programs offering well-being training	Percentage offered >90%
	Employers should provide resilience and well-being programs to nurses	Online surveys of healthcare facilities	Proportion of healthcare facilities offering well-being training	Percentage offered >75%
	Employees should provide a safe environment	Online assessment of nursing perception of work environment	Need to develop a Likert-score questionaries to measure cultural safety	Percentage staff feeling safe > 75%
Empowering nurses by changing policies and payment mechanisms	Change CPT codes to reimburse nurses for care coordination, team-based care, school nursing and teleservices	CPT codes are publicly available by CMS	Utilization of specific CPT codes	Increased utilization by > 100% from baseline
	Make permanent all COVID-19 nursing scope expansions	Assessment of state regulations for nursing practice	Number of states with permanent expansion of nursing scope	Increase the number of states to 45 (90%)
Improving the quality and accessibility of nursing education	Nursing programs should add SDOH competencies to their curricula	Online survey of nursing schools	Percentage of schools with SDOH competencies incorporated	Percentage offered >75%
	Nursing schools should provide distance learning opportunities		Percentage of schools offering distance learning	Percentage offered >50%
	Nursing programs should increase diversity among their faculty		Number of minority staff in faculty	Increase by 50% from baseline
	Nursing schools should encourage student civic engagement		Percentage of schools with policies promoting civic engagement	Percentage offered >50%
Developing a robust public health emergencies preparedness response plan	CDC to develop a nursing hub for nursing disaster preparedness response	CDC external communication	Development of the nursing hub	Hub established
	Nursing schools should incorporate emergency preparedness in curricula	Online surveys of nursing programs	Percentage of schools offering emergency preparedness skills	Percentage offered >75%
	Nursing boards should incorporate emergency preparedness in their licensing exams	Online surveys of nursing boards	Percentage of nursing boards requiring emergency preparedness as part of their licensing exams	Percentage offered >75%
	Healthcare systems should include nurses in their emergency preparedness plans	Online surveys of healthcare facilities	Percentage of healthcare facilities with nursing representation in their emergency plans	Percentage with representation > 90%
Including nursing expertise in technology design and implementation	Employers should include nurses with technology expertise in their EHR deployment teams	Online surveys of healthcare facilities	Percentage of healthcare facilities with nurses included in their EHR teams	Percentage > 75%
	EHR should capture SDOH data		Percentage of healthcare facilities with EHR features capturing SDOH	Percentage > 50%
Long term (by 2026)				
Strengthening the nursing role in advancing equity	Increase the number of nurses with health equity expertise	Online surveys of healthcare facilities	Number of nurses with specific health equity training	Increase from baseline by 100%
	Increase the number of nurses in shortage areas	Online surveys of healthcare facilities	Number of specialized nurses in specific areas	Increase from baseline by 100%
	Develop state programs to advance students from disadvantaged socioeconomic status	Online surveys of nursing schools	Percentage of schools with established policies promoting advancement	Percentage > 75%
	Include nursing expertise during state health reforms	Assessment of state regulations	Percentage of states requiring nursing presence in health reforms	Percentage > 50%
Investing in relevant research	Develop nursing grants to fund priority nursing research	Online surveys of nursing schools	Amount of funding for nursing research	Increase by > 50% from baseline

Abbreviations: CMS, Centers for Medicare and Medicaid Services; COVID-19, coronavirus disease 2019; CPT, current procedure terminology, EHR, electronic health records; SDOH, social determinants of health.

Source: Data adapted from 6 20 27 28

### Short-Term Recommendations (by 2024)

(1) Investing in the health and well-being of nurses: Focusing on nursing health and well-being should be part of nursing schools and health organizations. Employers should provide an environment that is both physically and physiologically safe (e.g., available personal protective equipment [PPE] and no retaliation), support diversity, and include nurses in key organizational decisions.
(2) Empowering nurses by changing policies and payment mechanisms: All temporary COVID-19 nursing scope expansions should be made permanent, including telehealth and insurance coverage policies. Payment models should be restructured to allow reimbursement of nurses for care coordination, case management, telehealth, and school nursing.
(3) Improving the quality and accessibility of nursing education: Programs should provide students with knowledge and skills to address equities, provide distance learning opportunities, promote a diverse faculty with experience in SDOH, and encourage civic engagement.
(4) Developing a robust public health emergency preparedness response plan: A national nursing hub 6 should be developed to build nursing education and staffing plans during emergencies. School curriculum and licensing exams should emphasize pandemic preparedness. Health care systems should include nursing in their local emergency planning design and implementation.
(5) Including nursing expertise in technology design and implementation: A technology infrastructure should be created to capture the community knowledge and SDOH visualization. Nurses should be incorporated into innovation, optimizing person-centered care, care coordination, and improving equities.

### Long-Term Recommendations (by 2026)

(6) Strengthening the nursing role in advancing equity: Substantial actions should be taken to increase the number of nurses with a special focus on health equity expertise and specialties with marked shortages (e.g., mental health, geriatrics, maternal health, and school health). This will require investing in nursing education, collaborating with historically Black and Hispanic-serving universities, supporting student loans and scholarships, enabling students from disadvantaged backgrounds, and integrating nursing expertise during health reform planning.
(7) Investing in relevant research: Government funding should increase to strengthen evidence-based nursing research as a significant focus. Research priorities should include the nursing workforce, public health collaboration, improving equities, performance and outcome measures, improving diversity, nursing well-being, eliminating structural racism, restructuring payment models, disaster preparedness, and advancing technologies.

## Conclusions

In conclusion, there are several root causes for the gap between the nursing workforce and population needs. Addressing these causes requires better responding to the market demand and supply forces, understanding the population's needs, preparing a competent nursing workforce, optimizing services, technological innovation, funding research, and leadership transformation.
